# The Anti-Wrinkle Mechanism of Melatonin in UVB Treated HaCaT Keratinocytes and Hairless Mice via Inhibition of ROS and Sonic Hedgehog Mediated Inflammatory Proteins

**DOI:** 10.3390/ijms19071995

**Published:** 2018-07-08

**Authors:** Eun Kyung Park, Hyo-Jung Lee, Hyemin Lee, Ju-Ha Kim, Jisung Hwang, Ja Il Koo, Sung-Hoon Kim

**Affiliations:** College of Korean Medicine, Kyung Hee University, Seoul 02447, Korea; friendly124@hanmail.net (E.K.P.); hyonice77@naver.com (H.-J.L.); glansy555@gmail.com (H.L.); 964juha@hanmail.net (J.-H.K.); hjsung0103@naver.com (J.H.); freelink78@naver.com (J.I.K.)

**Keywords:** melatonin, UVB, HaCaT keratinocytes, collagen, ROS, hedgehog, Cox-2, MMP-1

## Abstract

Though melatonin is known to improve ultraviolet B (UVB)-induced oxidative damage and inflammatory conditions via the blockade of the nuclear factor (NF)-κB, interleukin (IL)-6, there is no report on the anti-wrinkle effect of melatonin to date. Hence in the present study, the anti-wrinkle mechanism of melatonin was elucidated in UVB treated HaCaT keratinocytes and hairless mice. Herein melatonin protected against a radical initiator tert-Butyl hydroperoxide (t-BOOH) induced reactive oxygen species (ROS) production, matrix metalloprotease 1 (MMP-1), pro-collagen and cytotoxicity in HaCaT keratinocytes. Additionally, melatonin suppressed the expression of sonic hedgehog (SHH) and GLI1 for hedgehog signaling and p-NF-κB, cyclooxygenase (COX-2), phospho-extracellular signal-regulated kinase-1 (p-ERK) for inflammatory responses in UVB treated HaCaT keratinocytes. Furthermore, melatonin protected skin from wrinkle formation, transdermal water loss in hairless mice irradiated by UVB for 8 weeks. Notably, melatonin prevented against epidermal thickness and dermal collagen degradation in UVB irradiated hairless mice by Hematoxylin and Eosin and Masson’s trichrome staining. Taken together, these findings suggest that melatonin reduces wrinkle formation via inhibition of ROS/SHH and inflammatory proteins such as NF-κB/COX-2/ERK/MMP1.

## 1. Introduction

Facial wrinkles are known as one of the most prominent features for skin aging [1]. Skin aging is usually classified as either intrinsic (chronological) and extrinsic (photoaging) aging. Extrinsic aging shows photoaged skin with rough wrinkles mainly due to exposure to sunlight including ultraviolet B, while intrinsic aging is induced by aging genetic factors with a pale and smooth wrinkle formation [2]. It is well documented that the loss of skin elasticity and degradation of elastic fibers and collagen are usually shown in aged or wrinkled skin [3,4]. Previous evidence reveals that reactive oxygen species (ROS) in dermal fibroblast and epidermal keratinocyte produce matrix metalloproteinases (MMPs) to induce collagen degradation leading to skin aging, since MMPs degrade the extracellular matrix, including collagen fibers and thus contributes to wrinkle formation [5]. Marked loss of fibrillin-positive structures such as collagen type IV, VII and XVII in dermal and epidermal junctions contribute to wrinkles by weakening the bond between the dermis and epidermis [6,7]. In particular, UVB exposure upregulates the expression of MMP-1, -3 and -9 as well as sonic hedgehog (SHH) and GLI1 [8,9] in the human epidermis to induce wrinkle formation. Thus, recently many phytochemicals such as magnolo l [10], abietic acid [11], Galla Chinensis extract [12] and green tea polyphenols [13] were reported to block skin damage due to chronic UVB irradiation. Additionally, melatonin was reported to protect human keratinocytes from UVB-induced oxidative stress and DNA damage. Nevertheless, the anti-wrinkle mechanism of melatonin still remains unclear to date. Thus, in the present study, the anti-wrinkle molecular mechanism of melatonin was investigated in t-BOOH or UVB treated human keratinocytes and UVB irradiated SKH-1 hairless mice.

## 2. Results

### 2.1. Melatonin Protected Against t-BOOH Induced ROS Production and Cytotoxicity in HaCaT Keratinocytes

**Figure 1:**
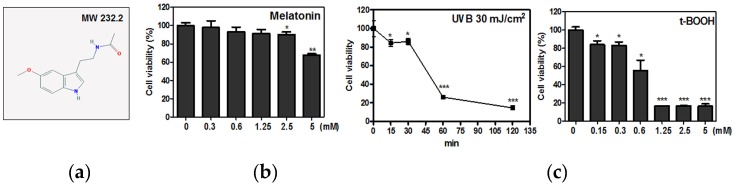
The effect of Melatonin, t-BOOH and UVB on the cytotoxicity in HaCaT cells. (**a**) Chemical structure of melatonin. Molecular weight = 232.2 (**b**,**c**) HaCaT cells were seeded onto 96-well microplates at a density of 1 × 10^4^ cells/well and treated with various concentrations of Melatonin (0, 0.3, 0.6, 1.25, 2.5 or 5 mM) or t-BOOH (0, 0.15, 0.3, 0.6, 1.25, 2.5 or 5 mM) for 24 h. HaCaT cells were exposed to UVB (30 mJ/cm^2^) for 15 min, 30 min, 60 min or 120 min. Data represent means ± SD. * *p* < 0.05, ** *p* < 0.01 and *** *p* < 0.001 versus untreated control.

**Figure 2:**
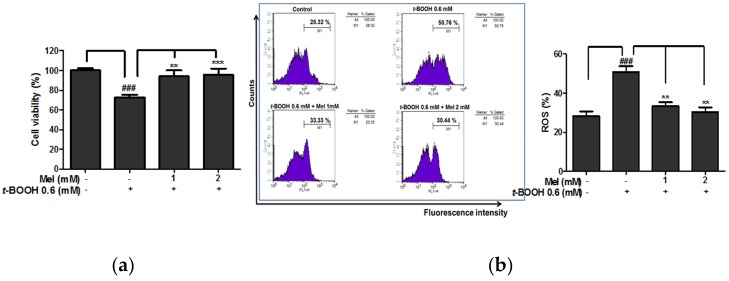
Melatonin suppressed t-BOOH induced cytotoxicity and ROS production in HaCaT keratinocytes. (**a**) Cells were pretreated with melatonin for 24 h and then with t-BOOH (0.6 mM) for 6 h. (**b**) ROS generation (%) was measured using ROS-sensitive fluorometric probe 2,7-dichlorofluorescein diacetate (DCFDA) by flow cytometric analysis. Data represent means ± SD. *** *p* < 0.001 and ** *p* < 0.01 versus t-BOOH control, ### *p* < 0.001 versus normal control.

It is well known that tert-Butyl hydroperoxide (t-BOOH) has been used as a radical polymerization initiator to induce cell damage [14]. To find out the protective effect of melatonin (Figure 1a) in t-BOOH induced cytotoxicity in HaCaT keratinocytes, an MTT assay was conducted. As shown in Figure 1b,c, t-BOOH or UVB (30 mJ/cm^2^) exerted cytotoxicity better than melatonin in HaCaT keratinocytes. However, melatonin significantly suppressed the t-BOOH induced cytotoxicity (Figure 2a) and ROS production at the concentrations of 1 and 2 mM in HaCaT keratinocytes (Figure 2b).

### 2.2. Melatonin Attenuated the mRNA Expression of MMP-1, Pro-Collagen Degradation in t-BOOH or UVB Induced HaCaT Keratinocytes

**Figure 3:**
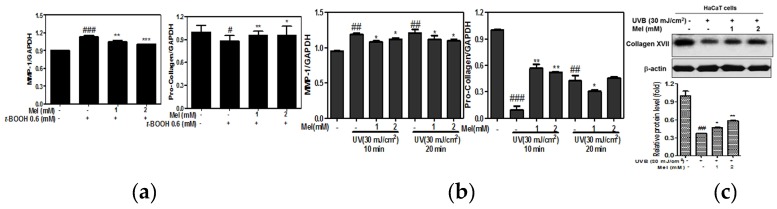
The melatonin attenuated the mRNA expression of MMP-1 and pro-collagen in t-BOOH or UVB induced HaCaT keratinocytes. (**a**) Cells were pretreated with melatonin for 24 h and then with t-BOOH (0.6 mM) for 6 h. mRNA expressions of MMP-1 and pro-collagen were analyzed by Real-Time PCR. Data represent means ± SD. *** *p* < 0.001, ** *p* < 0.01 and * *p* < 0.05 versus t-BOOH control, ### *p* < 0.001 versus normal control. (**b**) Cells were pretreated with melatonin for 24 h and then irradiated by UVB (30 mJ/cm^2^) for 10 min or 20 min. mRNA expressions of MMP-1 and pro-collagen were analyzed by Real-Time PCR. Data represent means ± SD. ** *p* < 0.01 and * *p* < 0.05 versus UVB control, ### *p* < 0.001 and ## *p* < 0.01versus normal control. (**c**) HaCaT cells were exposed to UVB (30 mJ/cm^2^) for 10 min and subjected to Western blotting for collagen XVII and β-actin. Graphs represent quantitative data of protein expression adjusted by β-actin. Data represent means ± SD. ** *p* < 0.01 and * *p* < 0.05 versus UVB control, ## *p* < 0.01 versus normal control.

There is accumulating evidence that the expression of MMPs was upregulated in t-BOOH induced HaCaT keratinocytes [11,15]. Additionally, UVB-induced photoaging is known to be mediated by increased expression of MMP-1 and collagen degradation [16,17]. To determine the effects of t-BOOH or UVB on MMP-1 expression, a qRT-PCR assay was performed. As shown in Figure 3a, melatonin reduced the mRNA expression of MMP-1 and increased the expression of pro-collagen in t-BOOH treated HaCaT keratinocytes. Additionally, melatonin suppressed the mRNA expression of MMP-1 and increased the expression of pro-collagen in UVB treated HaCaT keratinocytes (Figure 3b). Additionally, melatonin increased the expression of collagen XVII in UVB treated HaCaT keratinocytes (Figure 3c).

### 2.3. Melatonin Effectively Suppressed the UVB-Induced Hedgehog Signaling and MMP-1 Related Proteins in HaCaT Keratinocytes 

**Figure 4:**
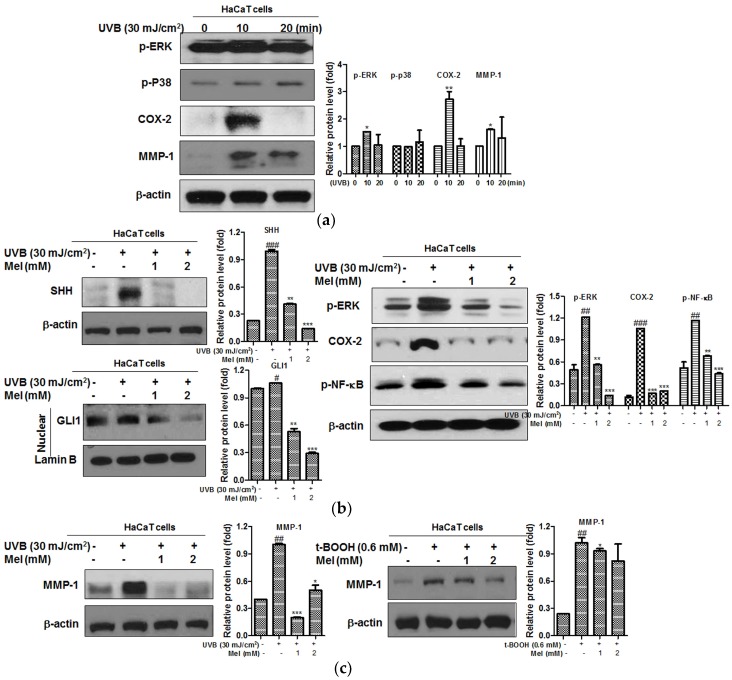
The melatonin attenuated the expression of SHH, GLI1 and MMP-1 related proteins in t-BOOH or UVB treated HaCaT keratinocytes. (**a**) HaCaT cells were exposed to UVB (30 mJ/cm^2^) for 10 min or 20 min and subjected to Western blotting for p-ERK, PP38, COX-2, MMP-1 and β-actin. (**b)**, (**c**) Cells were pretreated with melatonin for 24 h, irradiated with UVB (30 mJ/cm^2^) for 10 min and then subjected to Western blotting for SHH, GLI1 (nuclear fraction), p-ERK, COX-2, p-NF-κB, MMP-1, β-actin and Lamin B. Cells were pretreated with melatonin for 24 h and then with t-BOOH (0.6 mM) for 6 h. Graphs represent quantitative analysis of protein expression adjusted by β-actin. Data represent means ± SD. *** *p* < 0.001, ** *p* < 0.01 and * *p* < 0.05 versus UVB control, ### *p* < 0.001, ## *p* < 0.01 and # *p* < 0.05 versus normal control.

To determine whether melatonin inhibits UVB-induced MMP-1(photoaging marker) related proteins, Western blotting was performed. After the exposure to UVB 30 mJ/cm^2^ for 10 min or 20 min, MMP-1 related proteins (p-ERK, pp-38, COX-2 and MMP-1) were significantly affected at UVB 30 mJ/cm^2^ for 10 min (Figure 4a). To determine the molecular mechanism of melatonin, we examined the effects of melatonin on the hedgehog signaling pathway, since hedgehog signaling (SHH/GLI1) plays a key role in inducing MMP-1 [18]. Consistently, melatonin reduced the expression of UVB-induced SHH and GLI1 at 1 and 2 mM (Figure 4b). Additionally, melatonin reduced the expression of p-NF-κB, p-ERK and COX-2 in the UVB-treated HaCaT keratinocytes, while the phosphorylation of NF-κB, ERK and COX-2 was increased by UVB irradiation (Figure 4b). Furthermore, melatonin suppressed the expression of MMP-1 in t-BOOH or UVB treated HaCaT keratinocytes (Figure 4c).

### 2.4. Melatonin Prevented Against Loss of Water on the Dorsal skin of SKH1 Hairless Mice Exposed to UVB

**Figure 5:**
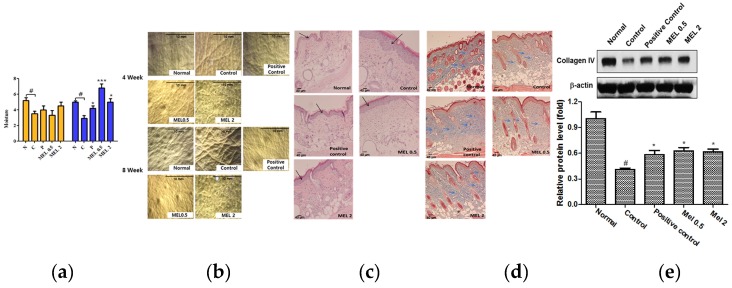
The melatonin significantly suppresses wrinkles and collagen fibers in the dorsal skin of SKH 1 hairless mice. N, Normal; C, Control + UVB irradiation; P, Positive control: Retinoic acid 2 mg/kg + UVB; MEL 0.5, Melatonin 0.5 mg/kg + UVB; MEL 2, Melatonin 2 mg/kg + UVB. (**a**) Effect of melatonin on water loss in the dorsal skin of SKH 1 hairless mice. Data represent means ± SD. *** *p* < 0.001 and * *p* < 0.05 versus UVB Control, # *p* < 0.05 versus Normal control. (**b**) Effect of melatonin on wrinkles in the dorsal skin of SKH 1 hairless mice. Melatonin reduced the degree of wrinkles induced by UVB irradiation on the dorsal skin of SKH1 hairless mice. Images of skin replicas were casted on the dorsal skin surface of mice using SILFLO. Scale bars = 10 mm (**c**) Hairless mouse skin was stained with hematoxylin and eosin (×100). Epidermis thickness was measured under light microscopy. Black arrow (epidermis). Scale bars = 40 μm (**d**) Collagen fiber formation was evaluated using Masson's trichrome staining. Collagen fiber bundles are shown in blue area (Blue arrow) (×100) Scale bars = 40 μm. (**e**) Effect of melatonin on collagen IV expression in UVB-exposed mice skin tissues. Skin tissues were homogenized on ice and the extracts were subjected to Western blotting. Graphs represent the quantitative analysis of protein expression adjusted by β-actin. Data represent means ± SD. * *p* < 0.05 versus UVB control, # *p* < 0.05 versus normal control.

It is well known that UVB can induce skin damages such as melanogenesis, erythema and epidermal thickness in the epidermis, leading to wrinkle formation in the dermis [19]. As shown in Figure 5a, melatonin significantly reduced the loss of water on the dorsal skin of SKH-1 hairless mice 8 weeks after UVB treatments, while the loss of water was accentuated in the UVB alone treated control compared to untreated normal control.

### 2.5. Melatonin Suppressed the Degree of Wrinkles and Epidermal Thickness Induced by UVB Irradiation on the Dorsal skin of SKH1 Hairless Mice

The degrees of wrinkles were observed on the dorsal skin of UVB irradiated SKH-1 hairless mice by SILFLO casting method, while those were reduced in melatonin- treated SKH-1 hairless mice under UVB exposure are shown in Figure 5b. Consistently, melatonin treatment reduced epidermal thickness induced by UVB irradiation by H&E staining and Masson’s trichrome staining revealed that melatonin treatment maintained the blue color for collagen staining compared to the UVB- alone- treated control in the dermis of the skin of UVB irradiated SKH-1 hairless mice as shown in Figure 5c,d. Consistently, as shown in Figure 5e, melatonin significantly blocked the degradation of collagen IV in skin tissues of SKH-1 hairless mice exposed to UVB irradiation.

## 3. Discussion

Melatonin (*N*-acetyl-5-methoxytryptamine) is known to have multi-biological activities to regulate circadian rhythms [20] and to work as a hormone or neurotransmitter [21], and as an anti-inflammatory [22,23] and antioxidant [24] agent. Especially, melatonin suppressed the ROS production in rat lens [25] and IL-3 stimulated leukocytes [24] and also enhanced the survival of UVB treated keratinocytes by suppressing apoptosis [26]. 

Herein, the anti-wrinkle mechanism of melatonin was elucidated in t-BOOH or UVB treated HaCaT keratinocytes and UVB treated hairless mice. 

Melatonin suppressed the ROS production and cytotoxicity in free radical initiator t-BOOH treated HaCaT keratinocytes, implying that melatonin protects HaCaT keratinocytes from t-BOOH induced cytotoxicity, since ROS play a pivotal role in skin damage by UVB-irradiation. 

UVB-induced photoaging is initiated by the production of ROS [27,28], which activate several receptors for IL-1, keratinocyte growth factor and tumor necrosis factor (TNF)-α [29,30]. Activation of these receptors induces downstream signaling pathways of mitogen-activated protein kinases (MAPKs) such as p38, JNK and ERK, which lead to an increase of the nuclear transcription such as NF-κB and AP-1 [29,31,32]. Additionally, the activation of the transcription factor NF-κB induces the expression of inflammatory cytokines such as COX-2, IL-6 and MMPs [33,34]. Continuous UVB exposure induces expression of MMPs, which degrades collagen by collagen breakdown as well as by the inhibition of procollagen synthesis [35,36]. Interestingly, melatonin suppressed the expression of COX-2, p-ERK, p-NF-κB and MMP-1 and increased the expression of procollagen and collagen XVII in UVB induced HaCaT cells, indicating that melatonin inhibits inflammatory cytokines and photoaging markers, leading to an anti-wrinkle effect in the skin.

The accumulating evidence reveals that the Hedgehog (Hh) signaling pathway regulates normal growth but its abnormal activation can promote cancer progression and epidermal development [8,9,37,38]. Therefore, to identify the molecular mechanism associated with UVB-induced photoaging, the role of Hh signaling was evaluated in UVB treated HaCaT cells. Here, melatonin reduced the expression of UVB-induced SHH as a Hh ligand and GLI1 as a transcriptional effector of Hh pathway, demonstrating that melatonin exerts an anti-photoaging effect via the inhibited Hh signaling pathway.

It is well documented that UVB induces skin damages leading to wrinkle formation due to collagen degradation in the dermis [39,40]. Here, melatonin protected skin from wrinkle formation, transdermal water loss in UVB treated hairless mice comparable to positive control retinoic acid, indicating that melatonin works against wrinkle formation in the dermis possibly via inhibition of collagen degradation. Consistently, Masson’s trichrome staining revealed that melatonin treatment maintained the blue color staining for collagen and reduced the epidermal thickness compared to the UVB- alone-treated group in the dermis of the skin of UVB irradiated SKH-1 hairless mice, implying a protective effect of melatonin on wrinkle formation by collagen degradation. 

Collectively, melatonin suppressed ROS production and the expression of MMP-1 in t-BOOH-induced HaCaT keratinocytes. Additionally, melatonin suppressed the expression of UVB-induced MMP-1 and MMP-1 related proteins (inflammatory proteins), increased procollagen in HaCaT keratinocytes and reduced the expression of hedgehog signaling proteins and protected against epidermal thickness, transdermal water loss, collagen degradation and wrinkle formation on the dorsal skin of UVB treated hairless mice. Overall, these findings demonstrate that melatonin protects against UVB induced oxidative skin damage including wrinkle formation via the inhibition of ROS, MMP-1 and the hedgehog signaling pathway.

## 4. Materials and Methods

### 4.1. Chemicals

Melatonin (*N*-acetyl-5-methoxytryptamine) (M5250) was purchased from Sigma (St. Louis, MO, USA). 

### 4.2. Cell Culture

Human skin keratinocyte cell line HaCaT cells (CRL-2404) were bought from American Type Culture Collection (ATCC). The cells were grown in Dulbecco Modified Eagle Medium (DMEM) (Gibco, Grand Island, NY, USA) with 10% fetal bovine serum, antimycotic (amphotericin B 0.25 μg/mL) and antibiotics (penicillin, 100 U/mL; streptomycin, 100 μg/mL). 

### 4.3. UVB Irradiation

HaCaT cells were treated with melatonin (0, 1 or 2 mM) for 24 h. After washing with phosphate-buffered saline (PBS), the cells were irradiated with 30 mJ/cm^2^ of UVB (312 nm) for 10 min using the CL-1000 Ultraviolet Crosslinker (Ultra-violet products Ltd, Cambridge, UK).

### 4.4. Cell Viability Assay

The cytotoxic effect of melatonin was assessed by using a 3-(4,5-dimethylthiazol-2-yl)-2,5-diphenyltetrazolium bromide (MTT) assay. In brief, HaCaT cells (1 × 10^4^ cells/well) were seeded onto 96-well culture plates and treated to various concentrations of melatonin for 24 h. The cells were incubated with MTT (1 mg/mL) (Sigma Chemical, St. Louis, MO, USA) for 2 h and the formazan crystals were dissolved in DMSO. The Optical density (OD) was measured by using a microplate reader (Molecular Devices Co., CA, USA) at 570 nm wavelength. Cell viability was calculated as a percentage of viable cells in melatonin-treated group versus untreated control.

### 4.5. RT-qPCR Assay 

RT-qPCR was performed with the LightCycler TM instrument (Roche Applied Sciences, Indianapolis, IN, USA) with the following primers, human MMP-1 forward: 5′-CATGACTTTCCTGGAATTGG-3′; reverse-5′-CCTGCAGTTGAACCAGCTAT-3′ (Bioneer, Daejeon, Korea), human pro-collagen forward: 5′-CAGGCAAACCTGGTGAACA-3′; reverse-5′-CTCGCCAGGGAAACCTCT-3′, human GAPDH-forward: 5′-GCACCGTCAAGGCTCTAGAAC-3′; reverse-5′-GGATCTCGCTCCTGGAAGAT-3′ (Bioneer, Daejeon, Korea). 

### 4.6. Western Blotting 

HaCaT Cells were pretreated with melatonin for 24 h, then with t-BOOH (0.6 mM) and exposed to UVB irradiation (30 mJ/cm^2^) were lysed in lysis solution (50 mM Tris–HCl, pH 7.4, 150 mM NaCl, 1 mM EDTA, 1 mM Na_3_VO_4_, 1% Triton X-100, 0.1% SDS, 1 mM NaF and 1× protease inhibitor cocktail) on ice and spun down at 14,000× *g* for 20 min at 4°C. The supernatants were collected and quantified for protein concentration by using an RC DC protein assay kit (Bio-Rad, Hercules, CA, USA), The proteins samples were separated on 4–12% NuPAGE Bis–Tris gels (Novex, Carlsbad, CA, USA) and transferred to a Hybond ECL transfer membrane for detection with antibodies for p-ERK, pp-38, COX-2, MMP-1, SHH and GLI1 (Cell Signaling Technology, Beverly, MA, USA), Collagen IV, Collagen XVII (Abcam, Cambridge, UK), Lamin B (Santa Cruz Biotechnologies, Santa Cruz, CA, USA) and β-actin (Sigma, St. Louis, MO, USA).

### 4.7. Nuclear Fraction for GLI1

HaCaT cells were pretreated with melatonin for 24 h and then exposed to UVB irradiation (30 mJ/cm^2^). Then nuclear fraction of the cells was prepared using NE-PER nuclear and cytoplasmic extraction reagents (Thermo, Rockford, IL, USA) based on the manufacturer’s protocol for Western blotting with the GLI1 antibody.

### 4.8. Measurement of Reactive Oxygen Species (ROS) Generation

The compound 2,7-Dichlorofluorescein diacetate (DCFH-DA) was used to measure the level of ROS production. HaCaT cells were pretreated with melatonin for 24 h, with t-BOOH (0.6 mM) for 6 h and then exposed to 5 μM DCFH-DA for 30 min at 37 °C. Fluorescence intensity was measured by FACSCalibur (Becton Dickinson, Franklin Lakes, NJ, USA).

### 4.9. Animals and Treatments

Fifty female SKH-1 mice (6-week-old) were purchased from Samtako Biokorea (Samtako Biokorea, Osan, Kyunggido, Korea) and housed at IK Science cage under consistent condition (temperature: 22 ± 1 °C, humidity: 55 ± 3%, 12 h light/ dark cycles). All experiments were performed in accordance with the guidelines established by Kyung Hee University. Mice were acclimated for one week and randomly divided into five groups (*n* = 10) during the experimental period: the normal group (N), control group (only UVB treated group; three times a week by UV-1000 group (Dongseo Science, Dangjin, Korea), positive control retinoic acid (Sigma Aldrich, USA) group (2 mg/kg) (B), melatonin 0.5 mg/kg group (MEL 0.5) and the melatonin 1 mg/kg group (MEL 1). Melatonin was orally administered to the mice of each group daily for 8 weeks, while saline was administered to the normal and control groups and retinoic acid was i.p injected in the positive control group three times a week for 8 weeks. 

**Figure 6:**
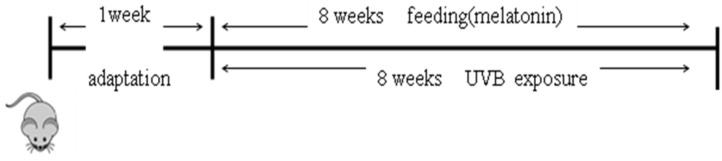
UVB irradiation schedule for SKH-1 hairless mice.

UVB irradiation schedule is as follows (Figure 6):

(1) 0 week: 60 mJ/cm^2^ for 200 s, three times a week (1 minimal erythema dose; M.E.D)
(2) 1 week: 120 mJ/cm^2^ for 400 s, three times a week (2 M.E.D)
(3) 2~3 weeks: 180 mJ/cm^2^ for 600 s 1 week: 120 mJ/cm^2^ for 400 s, three times a week (3 M.E.D)
(4) 4~5 weeks: 240 mJ/cm^2^ for 800 s, three times a week (4 M.E.D)

### 4.10. Observation of Skin Wrinkle Formation

The condition of skin wrinkle formation on the dorsal skin of hairless mice exposed to UVB irradiation was observed at 4 weeks and 8 weeks, the replicas were cast on the dorsal skin surface of the mice using SILFLO (Flexico developments LTD. Tokyo, Japan) and then photographed with a camera (Nikon D90, Tokyo, Japan).

### 4.11. Measurement of Skin Moisture

Mice were kept in the room in conditions of 21.5 ± 2 °C and relative humidity of 40 ± 5% for 30 min. Skin moisture was measured in melatonin-treated mice using Corneometer CM 820 (Courage-Khazake, Koln, Germany). 

### 4.12. Histological Analysis 

The dorsal skin was obtained from the melatonin or retinoic acid treated group and control group mice by biopsy, fixed in 4% paraformaldehyde, dehydrated in ethanol and then embedded in paraffin. Approximately 10 μm-thick sections were deparaffinized and stained with hematoxylin-eosin (H&E) and Masson’s trichrome staining. Stained slides were then photographed using a light microscope (ZEISS ObserverAD2, Gottingen, Germany).

### 4.13. Statistical Analysis

For statistical analysis of the data, the Sigmaplot version 12 software (Systat Software Inc., San Jose, CA, USA) was used. All data were expressed as means ± standard deviation (SD). A students *t*-test was used for the comparison of the two groups. The statistically significant differences were set at *p* values of <0.05 between the control and melatonin/retinoic acid treated groups.

## Abbreviations

UVB	Ultraviolet B
t-BOOH	*tert*-Butyl hydroperoxide
ROS	Reactive Oxygen Species
MMP-1	Matrix metalloprotease 1
mRNA	messenger RNA
PCR	Polymerase Chain Reaction
SHH	Sonic hedgehog
p-ERK	Phospho-Extracellular Signal-regulated Kinase-1
p-P38	Phospho -P38 mitogen-activated protein kinases
COX-2	Cyclooxygenase-2
i.p	intraperitoneal
NF-κB	Nuclear factor kappa-light-chain-enhancer of activated B cells
